# ShenQi FuZheng injection as an adjunctive treatment to chemotherapy in breast cancer patients: a meta-analysis

**DOI:** 10.1080/13880209.2019.1660383

**Published:** 2019-09-15

**Authors:** Hongbo Zhang, Tingting Chen, Lizhu Shan

**Affiliations:** Department of Medical Oncology, Tianjin Hospital of Integrated Traditional Chinese and Western Medicine, Nankai Hospital, Tianjin, China

**Keywords:** Shenqi Fuzheng injection, adjunctive treatment, chemotherapy, breast cancer, meta-analysis

## Abstract

**Context:** Shenqi FuZheng injection (SFI) has been suggested as a complementary treatment of chemotherapy in China. However, little is known about it in western countries.

**Objective:** This study assesses the clinical effect of SFI combined with chemotherapy for breast cancer patients.

**Materials and methods:** Both English and Chinese databases were searched covering the time period of 1999– 2018 for relevant studies comparing the effect of SFI plus chemotherapy treatment with chemotherapy alone in patients with breast cancer. Target outcomes concerning treatment effect, performance status, immune system and toxic effects were extracted and combined using Stata version 15.0 software. Quality assessment was performed using the Jadad scale.

**Results:** Forty-nine trials were included based on certain selection criteria. Only seven studies were rated as high-quality publications. Results of meta-analysis showed that SFI intervention can significantly improve objective tumour response, performance status, NK, CD_3_^+^, CD_4_^+^ and CD_4_^+^/CD_8_^+^ ratio and reduce occurrence of leucopenia, thrombocytopenia, haemoglobin reduction, liver dysfunction, gastrointestinal reaction, nausea and vomiting, bone marrow suppression and ECG changes. However, no significant difference was found between SFI and the control group regarding CD_8_^+^ levels, and renal disorders.

**Discussion and conclusions:** SFI intervention appeared to be effective in improving clinical efficacy, immune function and reducing toxicity when combined with chemotherapy for breast cancer. However, our findings still need verification by high-quality trials.

## Introduction

Breast cancer is one of the most prevalent cancers and the second leading cause of death among women. In 2019, 268,600 new breast cancer cases were estimated in US, accounting for approximately 30% of all new cases in women. Estimated deaths reached 41,760 cases, accounting for around 6.8% of all cancer-related death (Siegel et al. 2019). In China, more than 26,000 women were diagnosed and nearly 7000 died of breast cancer in 2015 (Chen, Zheng, et al. 2016). The predicted breast cancer mortality rate in 2020 was 13.4 per million patients (Carioli et al. 2017). Although the estimated mortality rate was lower than that in 2002–2012, the increasing number of new cases each year still made breast cancer an utmost important health care issue and economic burden worldwide (Montero et al. 2012; Tao et al. 2015). Thus, it is urgent for effective preventive and treatment for breast cancer.

Chemotherapy, surgery, radiotherapy, endocrine therapy, targeted therapy and immunotherapy are currently available treatment methods for breast cancer (Maughan et al. 2010; Peart 2015). However, the utility and success of these therapies are hampered by limitations such as timing, availability, cost and adverse side effects (Qi et al. 2015). Although the use of chemotherapy can achieve curative effect, the concomitant side effects and complications including myelosuppression, gastrointestinal reaction, haematological toxicity, cardiac damage may weaken treatment effect and lower survival rate of patients (Hutchins et al. 2005). Complementary and alternative medicines are introduced in this circumstance to relieve cancer symptoms and side effects, improve quality of life and survival in breast cancer patients (Leggett et al. 2015). Traditional Chinese medicine (TMC), which evolved over thousands of years with its own unique system, has been widely applied for cancer treatment in Asian countries, especially China.

Shenqi Fuzheng injection (SFI) is recommended for patients with malignant tumours to modify treatment effect since obtaining approval from the State Food and Drug Administration of the People’s Republic of China in 1999 (Li, et al. 2015a). It is comprised *Astragalus membranaceus* (Fisch.) Bunge (Fabaceae) and *Codonopsis pilosula* (Franch.) Nannf (Campanulaceae), which have been suggested to possess antimicrobial, antioxidant, antidiabetic and antitumour effects (Qi et al. 2015).

Accumulating evidence has suggested promising results of SFI in combination with conventional chemotherapy in improving success response rate, ameliorating patients’ quality of life, enhancing the immune function and reducing adverse events incidence (Dai et al. 2008; Lee et al. 2014; Jiang et al. 2015; Li, et al. 2015a; Lv et al. 2015; Liu et al. 2016). Nevertheless, many of these studies were restricted to Chinese population and little is known outside of China for lack of familiarity of the concept of SFI and doubt of the efficacy. In recent years, more and more attention has been focused on TCM. It was reported that about $120 million was spent each year on TCM related research projects in the United States National Cancer Institute (Li et al. 2013).

Thus, to promote TCM into the world and to provide more evidence of SFI in treating breast cancer, the present meta-analysis was performed in an effort to clarify whether SFI as adjunctive treatment to chemotherapy can increase objective tumour response, enhance immune function, reduce adverse side effects and eventually improve quality of life of breast cancer patients.

## Materials and methods

### Search strategy

To retrieve publications for meta-analysis, databases PubMed, ScienceDirect, China National Knowledge Infrastructure (CNKI), Wanfang, China Biological Medicine (CBM), were searched from 1999 (the year SFI was approved as medicine) to July 2018. The following search terms were used in databases searching: ‘breast cancer,’ ‘shenqi fuzheng,’ ‘chemotherapy,’ their abbreviations and all the synonyms adapted for each database. Language of study was restricted to English and Chinese. Reference lists of the potential articles and relevant review papers were also checked by the reviewers for additional studies.

### Inclusion and exclusion criteria

Publications meeting the following criteria were eligible for inclusion.

1. Studies were randomized controlled trials involving human subjects.
2. All the included patients were diagnosed with breast cancer according to the pathological, cytological and histological features regardless of disease stage and subtype.
3. Control group patients only received conventional chemotherapy as intervention. Patients in the experiment group received SFI plus the same chemotherapeutic drugs as the control group.
4. Studies compared patients’ outcome between SFI experiment and control group.
5. Studies must contain one or more of the following outcomes: objective tumour response, Karnofsky performance status, toxicity reaction, adverse side effects and immune function.
6. For studies which enrolled the same cohort of patients, only the newest version was included.

Studies that did not satisfy the above criteria, review articles, case reports, studies only in abstract form were all excluded from the meta-analysis. For relevant studies without sufficient data for calculation, we contacted the corresponding author for detailed information. If no response was received before the statistical analysis process, the study was excluded.

### Data extraction and quality assessment

Data were extracted by two reviewers independently based on a predefined form. Disagreements and discrepancies were resolved by discussion or the opinion of the third reviewer. All data, regarding study characteristics (author, year of publication and study design), patients demographics (sample size, gender, age, breast cancer subtype and stage), treatment and intervention (types of chemotherapy regimens, duration of treatment, doses and timing of the SFI intervention) and target outcomes, were extracted. For the present meta-analysis, outcomes of objective tumour response, performance status, immunity indicators including natural killer cell (NK), matured T lymphocytes (CD_3_^+^), inducer lymphocyte/helper T lymphocyte (CD_4_^+^), suppressor T cell/cytotoxic T cell (CD_8_^+^) and CD_4_^+^/CD_8_^+^ ratio, blood toxicity including white blood cells (WBC), haemoglobin (Hb) and platelet (PLT) and adverse events including nausea and vomiting, liver dysfunction, bone marrow suppression, renal disorder, gastrointestinal reaction and ECG changes were extracted.

A seven-point Jadad scale of randomized controlled trials was applied for quality assessment (Jadad et al. 1996). Study quality was evaluated based on six aspects, regarding randomization, allocation concealment, blinding, withdrawals and dropouts, inclusion/exclusion criteria and statistical analysis. Each study was scored from 0 to 7. Studies with scores of 4–7 were considered as high quality, whereas scores of 0– 3 represented poor or low quality.

### Statistical analysis

Meta-analysis was processed with Stata software version 15.0 (Stata Corporation, College Station, TX). For dichotomous data, pooled relative ratio (RR) with its 95% confidence intervals (CI) were calculated. A RR of more than one favoured the SFI intervention group in outcomes regarding tumour response and performance status. For blood toxicity and adverse side effects, a RR of less than one meant outcome in favour of the SFI treatment group. For continuous variables, weighted mean differences (WMD) with 95% CI was estimated when outcomes were measured with the same scale, and standardized mean differences (SMD) was utilized when different scales were used in different trials.

The overall heterogeneity of the enrolled studies was introduced by Q-statistic test and *I*^2^ test. The *I*^2^ value of 25–50%, 50–75%, or >75% were considered as low, moderate and high heterogeneity, respectively. A random-effect model was used for data calculation if *p* < 0.1 or *I*^2^ > 50%, otherwise, a fix effect model was applied (Deeks et al. 2011). Sensitivity analysis was performed by removing study outcome one by one to detect whether one particular study had dominant impact on the overall estimate and *I*^2^ value, and also to test the robustness of the study results (Iooss and Saltelli 2017).

Single-factor meta-regression analysis was applied to further evaluate the possible explanations for the heterogeneity. Studies were stratified into different subgroups based on chemotherapy regimen, timing of experiment and treatment duration. Variances were considered to be explanatory if their regression coefficients reached statistical significance (*p* < 0.05).

The risk of bias was estimated using the Cohrane Handbook of randomized controlled trials (Review Manager 5.3, The Cochrane Collaboration, 2014). Six items were assessed, and each components was judged on three levels (Yes, No and Unclear) (Higgins et al. 2011). Then, studies were categorized into low risk of bias (all the items were ranked as Yes), high risk of bias (at least one content were categorized No), and unclear risk of bias (at least one item was Unclear). Funnel plot and Begg’s and Egger’s test were generated to explore publication bias. *p* < 0.05 confirmed the existence of publication bias.

## Results

### Search results

**Figure 1.**
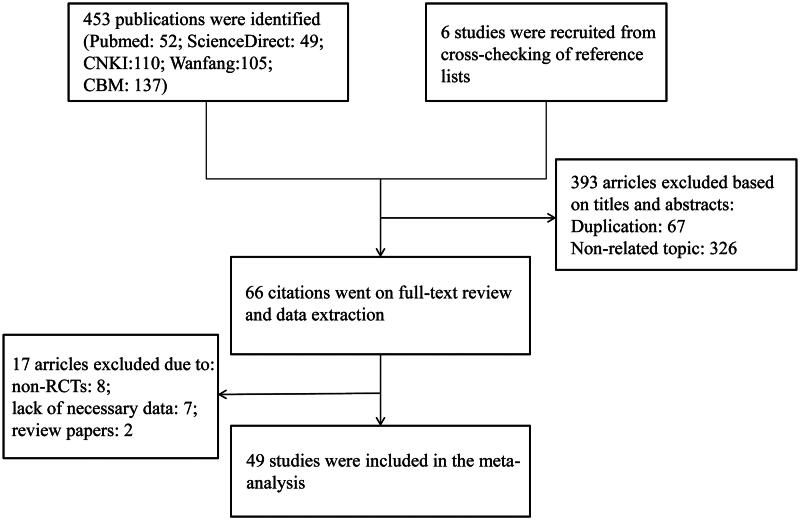
Flow diagram of literature search and selection.

A total of 453 records were identified from the English and Chinese databases at initial search. First screening of titles and abstracts was carried out by two reviewers, and 387 publications were excluded due to non-related topic and duplication. The remaining 66 citations went on full-text review and data extraction. Seventeen articles were further excluded for non-randomized controlled trials, lack of necessary data and review papers. Another six studies were recruited from cross-checking of reference lists; however, all six studies did not meet our inclusion criteria and were excluded. The final 49 studies were included in the meta-analysis (Li and Peng 2002; Li et al. 2004; Song 2004; Zhang 2004; Hong Wei 2005; Nie et al. 2005; Ren et al. 2005; Wang et al. 2006; Zhou et al. 2006; Chen and Li 2007; Dai et al. 2007; Yang et al. 2007; Huang et al. 2008; Yuan et al. 2008; Zhu et al. 2008; Gao and Xia 2009; Cha and Jia 2010; Chen et al. 2010; Lu et al. 2010; Qiu 2010; Xu and Yue 2010; Aticam and Akomatine 2011; Wu 2012; Qi et al. 2013; Qiao and Cui 2013; Shi et al. 2013; Wang 2013; Zhang et al. 2013; Feng 2014; Fu 2014; Li and Li 2014; Liang et al. 2014; Xie 2014; He 2015; Hong 2015; Li, et al. 2015b; Wang 2015a; Wang 2015b; Chen 2016; Chen, Gan, et al. 2016; Jia et al. 2016; Su and Zhou 2016; Wang et al. 2016; Xu and Xia 2016; Yang 2016; Liu and Song 2017; Liu 2017; Ou et al. 2017; Yu et al. 2017). The process of literature search and study selection are illustrated in Figure 1.

### Study characteristics

The included studies covered the period from 2002 to 2017, and were all conducted in China. A total of 4385 breast cancer patients (SFI intervention: 2229; controls: 2156) were enrolled, and patients’ age ranged from 18 to 70. Chemotherapy regimens used were varied. About 42.8% (21/49) of the included publications applied cyclophosphamide, adriamycin, 5-fluorouracil (CAF) regimen in chemotherapy, 12.2% (6/49) of trials used cyclophosphamide, pirarubicin, 5-fluorouracil (CTF) regimen, 10.2% (5/49) untilized cyclophosphamide, epirubicin, 5-fluorouracil (CEF), 6.1% (3/49) used cyclophosphamide, methotrexate, 5-fluorouracil (CMF) and 4.1% (2/49) applied gemcitabine, cisplatin (GP). In the experimental group, patients were given 250 ml of SFI daily through intravenous administration on the basis of chemotherapy. All SFI used in the included trials were manufactured by Livzon Pharmaceutical Group Inc., Zhuhai, China (approval number: Z19990065). Most of the studies (36/49) started SFI treatment at the beginning of chemotherapy and synchronized ended with chemotherapy. Seven trials started SFI treatment after the first or second cycle of conventional chemotherapy, and 6 studies started 1–3 d before chemotherapy. The detailed SFI treatment duration of each study was summarized in Table 1. Two studies reported using sham injection in the control group (Zhang 2004; Xie 2014), others applied conventional chemotherapy only. Of the 49 studies claimed to be RCTs, only 11 studies described clearly using the random digital table for randomization, and the others only stated that subjects were randomized without mentioning the specific methods or procedures. None of the included studies reported using a blinding method for data interpretation. Detailed information of each study can be found in Table 1.

**Table 1.** Basic characteristics of the included studies.

Author	Year	SFI (N)	Control (N)	Age (SFI)	Age (control)	Disease subtype	Disease stage	Shenqi Fuzheng injection	Duration of Fuzheng timing (days)	Chemotherapy	Jades
ATIKAN KAWULI	2011	40	40	28–65		–	Stage II/III/IV	250 ml intravenous	1 time/d, 63 d	TA	2
Cha and Jia	2010	31	31	35–60	32–61	–	Stage II/III/IV	250 ml intravenous	1 time/d, 14 d	Lifein 10 mg	4
Chen and Li	2007	34	34	38–64		IDC/ILC	Stage I/II	250 ml intravenous	3 d before CM; 1 time/d, 24 d	CEF	2
Chen et al.	2010	90	95	42	45	–	Stage III/IV	250 ml intravenous	3,5 d before CM; 1 time/d	TD	2
Chen	2016	42	42	42.65 ± 8.27	42.63 ± 8.24	IDC/ILC/DCIS	–	250 ml intravenous	1 time/d, 84 d	CAF	3
Chen, Gan et al.	2016	31	30	52.0 ± 11.45	50.43 ± 12.67	IDC/ILC	Stage I/II/III	250 ml intravenous	1 time/d, 5d per cycle	TEC	5
Dai et al.	2007	65	61	45.5 ± 26.8	46.1 + 27.5	–	LABC	250 ml intravenous	1 time/d, 56 d	CEF	2
Feng	2014	32	28	49.39 ± 5.72	51.18 ± 5.56	IDC	–	250 ml intravenous	3 d before CAF; 1 time/d, 126 d	CAF	3
Fu	2014	45	45	32–52		IDC/DCIS	–	250 ml intravenous	2 cycle after CAF; 1 time/d, 14 d	CAF	2
Gao and Xia	2009	32	31	35–65	33-68	IDC/ILC	LABC	250 ml intravenous	1 time/d, 14 d	NP	2
He	2015	38	38	41.75 ± 2.77	40.85 ± 5.12	–	–	250 ml intravenous	1 time/d, 14–147 d	CF	2
Hong	2015	25	25	44.42 ± 4.43	44.42 ± 4.43	–	LABC	250 ml intravenous	1 time/d, 147 d	GP	2
Huang et al.	2008	30	30	24–68	26–66	IDC	LABC	250 ml intravenous	1 time/d, 42 d	CTF	4
Jia et al.	2016	50	50	45.35 ± 10.02	43.78 ± 9.18	IDC	–	250 ml intravenous	1 cycle after CAF; 1 time/d, 14 d	CAF	2
Liang et al.	2014	27	27	45.8 ± 2.3	–	–	Stage III/IV	250 ml intravenous	1 time/d, 42 d	CTF	3
Liu	2017	80	80	46.21 ± 9.83	45.36 ± 3.37	IDC	–	250 ml intravenous	1 time/d, 21 d	CAF	4
Liu et al.	2016	52	52	41.58 ± 3.23	40.41 ± 3.72	IDC	–	250 ml intravenous	2 cycle after CAF; 1 time/d, 14 d	CAF	2
Li et al.	2004	40	35	56.4	54.2	IDC	LABC/Stage IV	250 ml intravenous	1 time/d, 10 d per cycle	NE	2
Li, Wang, et al.	2015	80	80	48.53 ± 5.17	48.42 ± 5.13	–	Stage II	250 ml intravenous	1 time/d, 126 d	CAF	4
Li and Li	2014	48	48	30–70	–	–	–	250 ml intravenous	3 d before CAF;1 time/d,10 d	CAF	2
Li and Peng	2002	35	27	47.2 ± 10.8	46.7 ± 10.5	–	–	250 ml intravenous	1 time/d, 63 d	DM	2
Lu et al.	2010	58	52	32–69	–	IDC	–	250 ml intravenous	2nd cycle day 2 of CAF; 1 time/d, 42–63 d	CAF	2
Nie et al.	2005	30	30	37–65	36–70	–	Stage IV	250 ml intravenous	beginning of every cycle; 1 time/d, 7 d	NVB	3
Qiao and Cui	2013	20	20	40–65	–	metastatic breast cancer	–	250 ml intravenous	1 time/d, 14 d	Capecitabine	2
Qi et al.	2013	26	20	52	–	IDC	Stage II/III/IV	250 ml intravenous	1 time/d, 21 d per cycle	CTF	2
Qiu	2010	24	23	34–65	–	IDC/ILC	Stage III/IV	250 ml intravenous	1 time/d, 14 d per cycle	PE	3
Qu et al.	2017	31	31	48.35 ± 12.23	48.02 ± 12.11	–	Stage II/III	250 ml intravenous	1 time/d,14 d	CAF	3
Ren et al.	2005	45	39	25–63	27–68	–	–	250 ml intravenous	1 time/d, 21 d per cycle	CAF	2
She et al.	2017	192	192	52.2 ± 8.62	52.7 ± 8.12	IDC/ILC	–	250 ml intravenous	1 time/d, 6 d	DE	4
Shi et al.	2013	34	34	45–61	47–65	IDC	–	250 ml intravenous	1 time/d; 1–14 d	NX	2
Song	2004	21	25	32–65	35–61	IDC/ILC	Stage II/III	250 ml intravenous	1 time/d, 28 d	CMF	1
Su and Zhou	2016	102	96	51.5 ± 7.8	52.5 ± 8.2	–	Stage II/III	Oral	2 time/d; 126 d	CTF	2
Wang	2013	38	38	45.5 ± 9.8	45.2 ± 9.8	IDC	Stage II/III	250 ml intravenous	2 week after CAF; 1 time/d, 14 d	CAF	3
Wang	2015a	47	45	42.85 ± 3.11	41.96 ± 3.88	IDC	Stage II/III	250 ml intravenous	2 week after CAF; 1 time/d, 14 d	CAF	2
Wang et al.	2006	40	32	45.2 ± 9.8	46.7 ± 10.5	IDC	–	250 ml intravenous	1 time/d, 126 d	CEF	3
Wang	2015b	65	65	46.3 ± 4.6	47.4 ± 5.2	IDC/ILC/DCIS	–	250 ml intravenous	1 time/d, 14 d	CAF	4
Wang et al.	2016	44	44	55.2 ± 6.1	55.5 ± 6.3	–	–	250 ml intravenous	1 time/d, 42–63 d	CAF	2
Wu	2012	36	36	51.5 ± 16.5	52.5 ± 16.5	–	–	250 ml intravenous	1 time/d, 112 d	CMF	2
Xiao	2005	55	53	43–67	–	–	–	250 ml intravenous	day 1–8; 1 time/d	CTF	3
Xie	2014	45	45	54.4 ± 3.8	52.8 ± 4.3	IDC/ILC/DCIS	–	250 ml intravenous	1 time/d, 42–63 d	CAF	3
Xu and Yue	2010	37	33	34–72	32–74	IDC/ILC	Stage II/III	250 ml intravenous	1 time/d, 35 d	CAF	2
Xu and Xia	2016	32	32	45.8 ± 5.2	–	IDC	Stage II/III	250 ml intravenous	1 time/d; 35 d	CAF	2
Yang et al.	2007	58	52	36–69	32–68	IDC	–	250 ml intravenous	2 week after CAF; 1 time/d, 14 d	CAF	2
Yang	2016	40	40	42.12 ± 1.33	42.89 ± 1.21	–	Stage I/II/III	250 ml intravenous	3 d before CAF;1 time/d, 10 d	CEF	2
Yuan et al.	2008	38	35	19–60	–	IDC/ILC	Stage II/III	250 ml intravenous	1 d before surgery; 1 time/d, 20 d	CAF	3
Zhang et al.	2013	32	32	43.2 ± 7.8	47 ± 6.5	–	Stage III	250 ml intravenous	1 time/d, 21 d	GP	3
Zhang	2004	28	27	–	–	–	–	250 ml intravenous	day 1–8; 1 time/d	CMF; CAF	3
Zhou et al.	2006	32	32	29–65	–	IDC/ILC	Stage II/III	250 ml intravenous	1 time/d, 14 d	CTF	2
Zhu et al.	2008	32	24	35–60	36–64	–	Stage I/II/III	250 ml intravenous	1 time/d, 10 d	CEF	2

IDC: invasive ductal carcinoma; ILC: invasive lobular carcinoma; DCIS: ductal carcinoma *in situ*; TA: paclitaxel, pirarubicin; CTF: cyclophosphamide, pirarubicin, 5-fluorouracil; TD: taxotere, pirarubicin; CAF: cyclophosphamide, adriamycin, 5-fluorouracil; TEC, cyclophosphamide, docetaxel, epirubicin; CEF: cyclophosphamide, epirubicin, 5-fluorouracil; NP: vinorelbine, cisplatin; CF: cyclophosphamide, 5-fluorouracil; GP: gemcitabine, cisplatin; NE: vinorelbine, epirubicin; DM: mitomycin, cisplatin; NVB: vinorelbine; PE: paclitaxel, adriamycin; DE: docetaxel, epirubicin; NX: vinorelbine, capecitabine; CMF: cyclophosphamide, methotrexate, 5-fluorouracil.

### Quality assessment and risk of bias analysis

**Figure 2.**
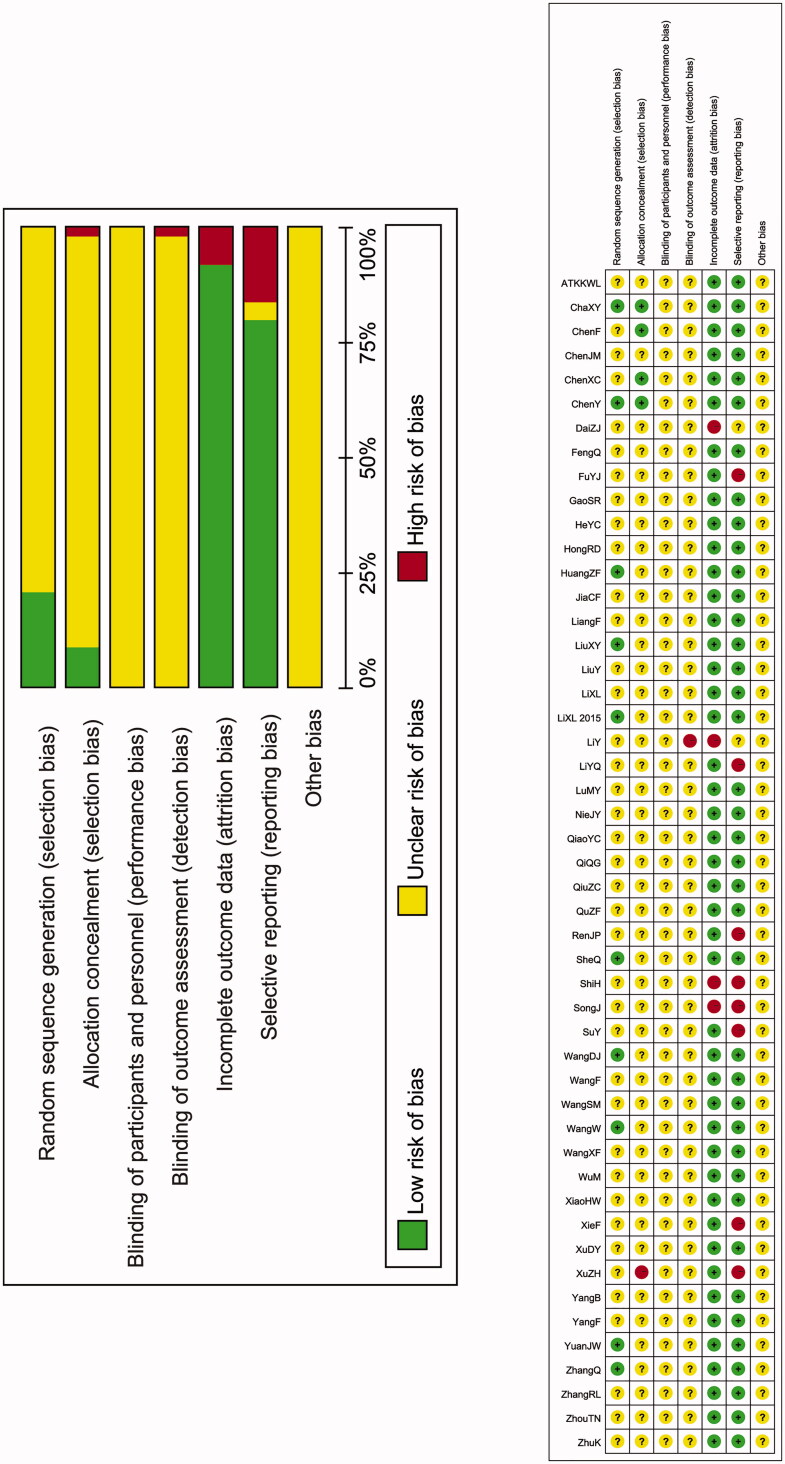
Risk of bias summary of 49 included studies.

According to the seven point Jadad scale assessment, only seven studies were considered as high quality as they reached the score of 4–5. Forty-two trials were ranked as low-quality studies as many of them did not describe a specific method for randomization or provide information regarding the blinding method, follow-up and inclusion/exclusion criteria. The total score of each study is presented in Table 1. Risk of bias analysis revealed that many of the included studies had unclear bias since whether or not researchers applied allocation concealment, blinding of participants and personnel, or blinding of outcome assessment were indeterminable based on the manuscript description. The results of risk of bias analysis and the distribution of studies were presented in Figure 2.

### Effect of SFI intervention on objective tumour response

**Figure 3.**
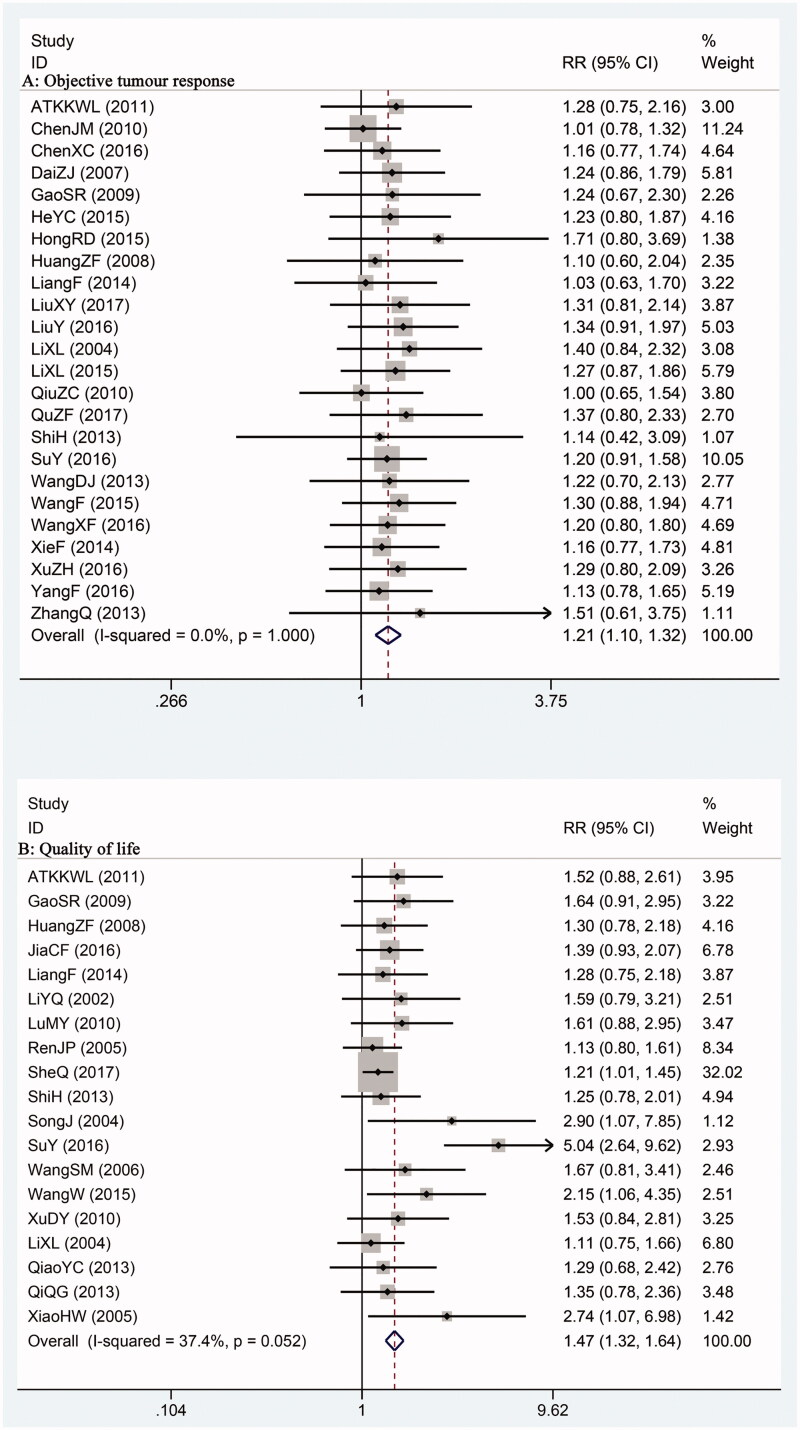
Comparison of objective tumor response rate (A) and performance status (B) between SFI intervention and control group.

Twenty-four trials provided outcome of overall response rate. Five studies used RECIST criteria to classify tumour response (Li et al. 2004; Gao and Xia 2009; Qiu 2010; Shi et al. 2013; Liu and Song 2017), and the rest used criteria suggested by UICC. Overall response rate was calculated combining complete response with partial response. Meta-analysis results showed that patients in SFI intervention group achieved better tumour response compared with control patients (RR = 1.21; 95% CI, 1.10–1.32) (Figure 3(A)). Sensitivity analysis did not find significant changes on both overall RR and *I*^2^ value when omitting each study result. Meta-regression analysis was also conducted to explore if chemotherapy regimen, timing of experiment, and treatment duration would bring heterogeneity. Results of meta-regression analysis showed that the *p* values of each subgroup were >0.05, meaning the three aforementioned factors were not the source of heterogeneity (Table 2).

**Table 2.** Results of regression meta-analysis.

	Chemotherapy regimen	Timing of experiment	Treatment duration
Objective tumour response			
Coefficient	0.023	0.038	0.014
Standard error	0.023	0.052	0.024
*t*	1.01	0.74	0.60
*p* Value	0.327	0.468	0.553
[95%CI]	−0.024 to 0.071	−0.070 to 0.147	−0.035 to 0.064
Performance status			
Coefficient	0.021	0.225	−0.214
Standard error	0.074	0.218	0.132
*t*	0.23	1.03	−1.61
*p* Value	0.825	0.319	0.127
[95%CI]	−0.180 to 0.229	−0.241 to 0.691	−0.496 to 0.068
CD3^+^			
Coefficient	−2.566	1.886	−2.334
Standard error	1.253	1.594	1.227
*t*	−2.05	1.18	−1.90
*p* Value	0.080	0.276	0.099
[95%CI]	−5.529 to 0.396	−1.885 to 5.657	−5.235 to 0.567
CD4^+^			
Coefficient	−0.077	0.727	−0.261
Standard error	0.382	0.477	0.351
*t*	−0.20	1.52	−0.75
*p* Value	0.844	0.158	0.473
[95%CI]	−0.928 to 0.774	−0.335 to 1.790	−0.335 to 0.519
CD8^+^			
Coefficient	−0.077	0.727	−0.261
Standard error	0.382	0.477	0.350
*t*	−0.20	1.52	−0.75
*p* Value	0.844	0.158	0.473
[95%CI]	−0.928 to 0.774	−0.335 to 1.790	−1.042 to 0.519
CD4^+^/CD8^+^			
Coefficient	−0.055	−0.014	−0.037
Standard error	0.024	0.029	0.021
*t*	−2.23	−0.48	−1.73
*p* Value	0.049	0.639	0.115
[95%CI]	−0.110 to −0.001	−0.079 to 0.051	−0.085 to 0.011
NK			
Coefficient	−4.417	3.609	−3.430
Standard error	4.467	4.129	2.244
*t*	−0.99	0.87	−1.53
*p* Value	0.361	0.416	0.177
[95%CI]	−15.349 to 6.515	−6.500 to 13.708	−8.922 to 2.061

### Effect of SFI intervention on performance status

Patients’ quality of life was evaluated in 19 studies by measuring Karnofsky performance score (KPS) (Karnofsky and Burchenal 1949). The rate of improved (KPS increase ≥ 10 points after treatment) or stable (KPS increase/decrease within 10 points after treatment) were compared between SFI and control group. Result demonstrated a favourable RR for SFI treatment (RR = 1.47; 95% CI, 1.32–1.64) (Figure 3(B)). Sensitivity analysis found that individual study did not significantly influence the overall result and heterogeneity. The results of meta-regression study also suggested that chemotherapy regimen, timing of experiment and treatment duration were not the source of heterogeneity (Table 2).

### Effect of SFI intervention on immune function

**Figure 4.**
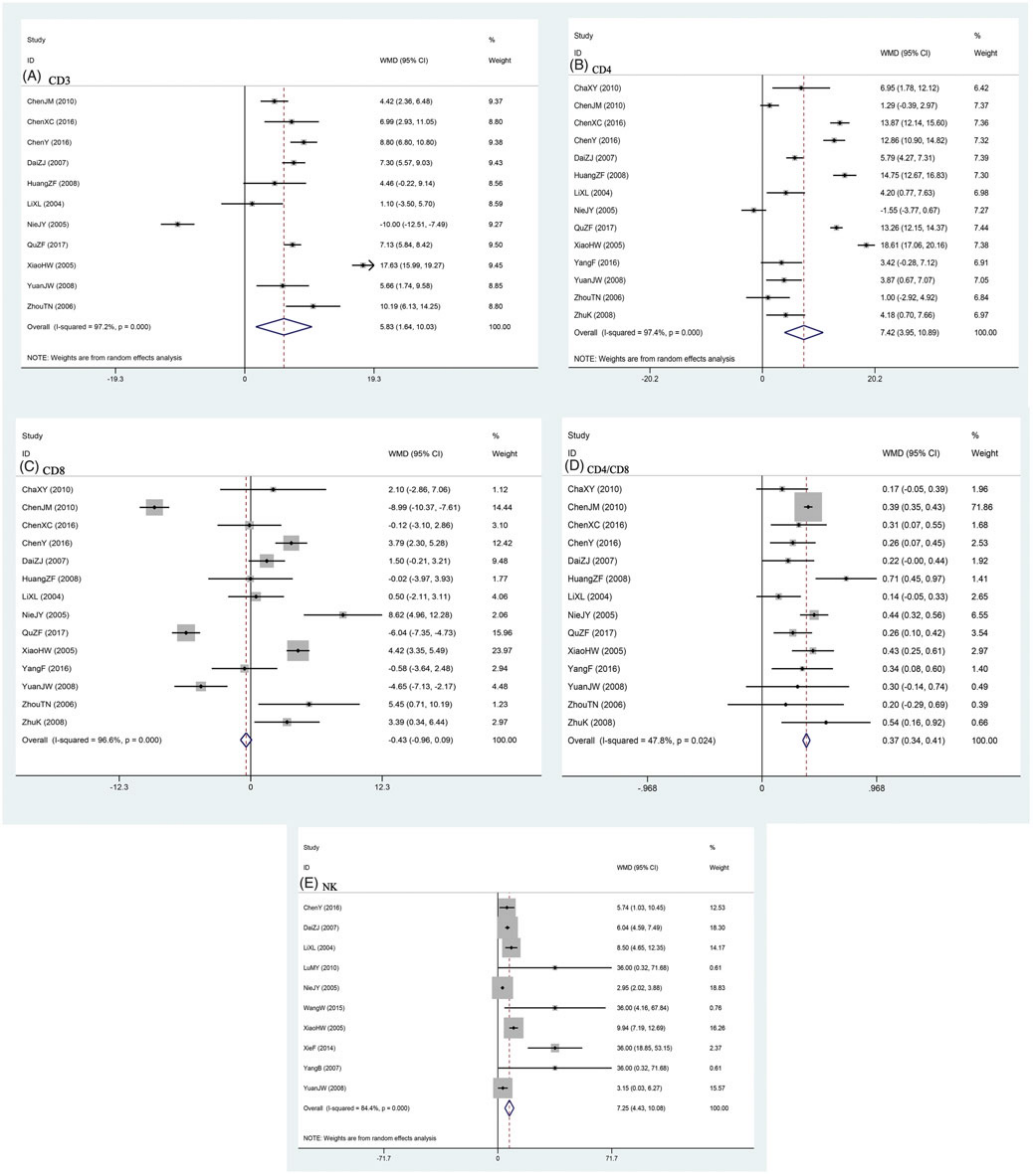
Comparison of immune function between SFI intervention and control group. A: CD_3_^+^; B: CD_4_^+^; C: CD_8_^+^; D: CD_4_^+^/CD_8_^+^ ratio; E: NK.

According to data from 11 trials, the CD3^+^ cell levels were significantly improved by SFI intervention (WMD = 5.83; 95% CI, 1.64–10.03) (Figure 4(A)). Due to considerable heterogeneity (*I*^2^=97.2%), sensitivity analysis was performed. Pooled WMD and *I*^2^ value did not change significantly compared with the overall result when removing each study result except the study of Nie. The removal of Nie et al. (2005) resulted in a decrease of *I*^2^ value from 97.2% to 66.7% while the overall effect remained statistically significant.

Summarized result of 14 studies showed that SFI intervention group was superior to the control group in increasing CD4^+^ expression (WMD = 7.42; 95% CI, 3.95–10.89) (Figure 4(B)). Considering high heterogeneity existed (I^2^ = 97.4%), sensitivity analysis was conducted. Results of sensitivity analysis also confirmed that the study of Nie affected the overall I^2^ value.

In 14 clinical trials concerning CD8^+^ levels, no statistical significance was found between SFI intervention and control group (WMD= −0.43, −0.96–0.09) (Figure 4(C)). Sensitivity analysis found the same result as the CD3^+^ and CD4^+^ subgroups.

Fourteen trials mentioned about CD4^+^/CD8^+^ ratio, combined result revealed that SFI intervention preceded the control group in improving CD4^+^/CD8^+^ ratio (WMD = 0.37, 0.34–0.41) (Figure 4(D)).

In 10 reports concerning NK cells in patients with breast cancer, results showed that NK levels were significant higher in SFI intervention group compared with controls (WMD = 7.25, 4.43–10.08) (Figure 4(E)). Sensitivity analysis did not find any opposite result when removing the included studies one by one.

Meta-regression analysis showed that chemotherapy regimen, timing of experiment and treatment duration did not have significant impact on CD3^+^, CD4^+^, CD8^+^, NK cell levels and CD4^+^/CD8^+^ ratio, except that chemotherapy regimen might be the heterogeneity source in CD4^+^/CD8^+^ ratio analysis (Table 2).

### Effect of SFI intervention on blood system

**Figure 5.**
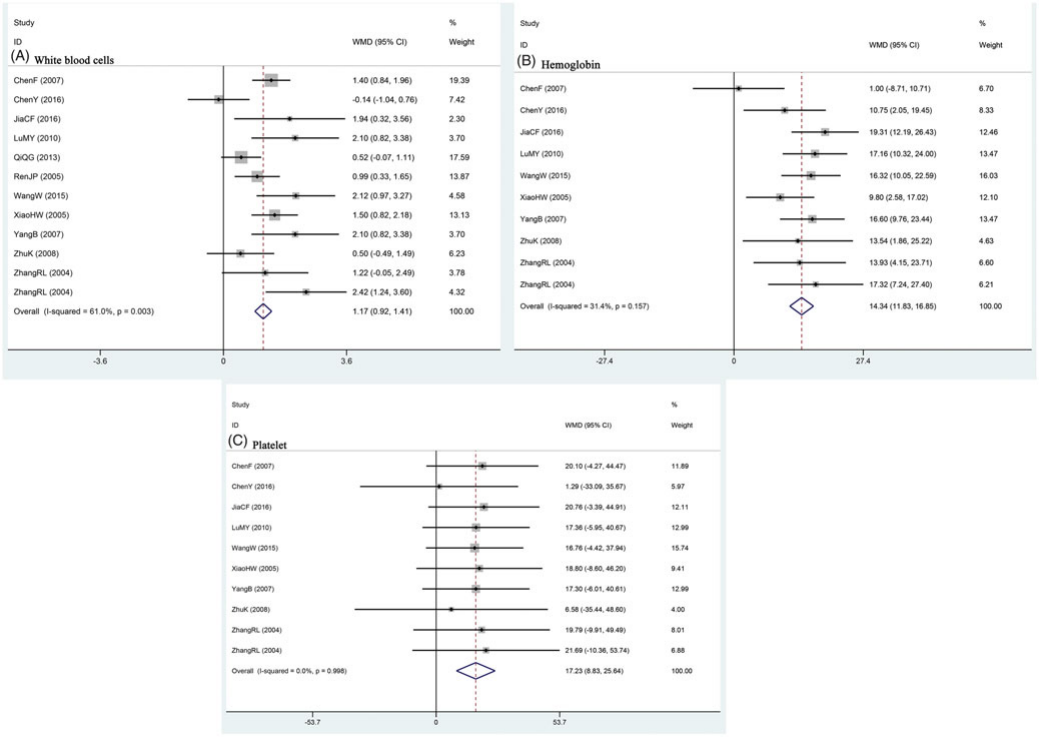
Comparison of blood system between SFI intervention and control group. A: white blood cells; B: haemoglobin; C: platelet.

The safety evaluation of WBC was reported in 11 studies and HB and PLT were measured in 9 trials, respectively. The study of Zhang (2004) applied two chemotherapy regimens and provided two groups of outcomes separately. Pooled WMD revealed that WBC, HB and PLT in patients receiving SFI intervention plus chemotherapy were significantly higher than those treated with chemotherapy alone (Figure 5).

### Effect of SFI intervention on toxicity and adverse side effects

Haematological toxicity was assessed by recording the incidence of leucopenia, thrombocytopenia and haemoglobin reduction. The RRs of leucopenia, thrombocytopenia and haemoglobin reduction were 0.76 (95% CI, 0.68–0.85), 0.73 (95% CI, 0.59–0.90) and 0.61 (95% CI, 0.43–0.87), respectively, indicating that SFI intervention had a lower risk of haematological toxicity compared with control group (Table 3).

**Table 3.** Efficacy of SFI intervention on blood system.

Toxicity and adverse side effects	N	RR	95% CI	*p*	*I*^2^-value (%)
Haematological toxicity					
Leucopenia	20	0.76	0.68, 0.85	<0.0001	9.8
Thrombocytopenia	10	0.73	0.59, 0.90	0.0003	0
Haemoglobin reduction	7	0.61	0.43, 0.87	<0.0001	0
Non-hematologic toxicity					
Liver dysfunction	11	0.50	0.37, 0.70	0.02	0
Gastrointestinal reaction	8	0.74	0.65, 0.85	<0.001	52.4
Renal disorder	3	0.57	0.17, 1.90	0.943	0
Nausea and vomiting	12	0.63	0.51, 0.77	<0.001	0
Bone marrow suppression	7	0.68	0.51, 0.91	<0.0001	0
ECG changes	7	0.65	0.51, 0.83	<0.0001	55.3

Non-haematologic toxicity regarding liver dysfunction (11 studies), renal disorder (3 studies) and gastrointestinal reaction (8 studies) were also evaluated. Results showed that SFI intervention could significantly reduce the damaging incidence of liver (RR = 0.50; 95% CI, 0.37–0.70) and gastrointestinal (RR = 0.74; 95% CI, 0.65–0.85) compared to conventional treatment alone. There was no significant difference between intervention and control group in the occurrence of renal disorder (RR = 0.57; 95% CI, 0.17, 1.90) (Table 3). However, only three studies were included in this subgroup and the results might be insufficient to draw conclusion.

Other side effects including nausea and vomiting (12 studies), bone marrow suppression (7 studies) and ECG changes (7 studies) were assessed. Pooled results demonstrated a low risk of nausea and vomiting (RR = 0.63; 95% CI, 0.51–0.77), bone marrow suppression (RR = 0.68; 95% CI, 0.51–0.91) and ECG changes (RR = 0.65; 95% CI, 0.51–0.83) in SFI intervention group when compared with control (Table 3).

### Evaluation of publication bias

**Figure 6.**
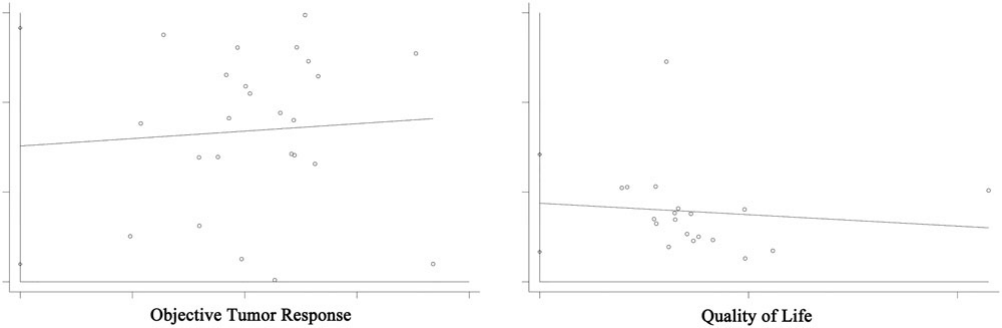
Publication bias of objective tumor response rate and performance status.

Begg’s and Egger’s test were performed based on studies with data on the objective tumour response and performance status. Both funnel plots and Begg’s and Egger’s tests (*p* < 0.05) suggested the existence of publication bias among the included studies (Figure 6).

## Discussion

TMCs have been developed alongside the Chinese history. In recent decades, the application of TCMs in treating malignant diseases is dramatically increasing. SFI is a formulation injection made from two Chinese medical materials Radix *Astragali* and *Codonopsis pilosula* with a rate of 1:1 (Zhuang et al. 2012; Wang et al. 2012). The two main ingredients of SFI have been widely applied to treat traumas such as animal bites, wounds and burns, small illnesses including poor appetite, fatigue and dyspepsia and deadly disease like cancer for thousands of years (Jin et al. 2008; Qi et al. 2008; Qian et al. 2010; Yang et al. 2013). With its antioxidant, antitumour and enhanced immune function ability, Chinese clinicians are confident in using SFI as adjunctive treatment for patients with breast cancer undergoing chemotherapy (Jin et al. 2008; Yang et al. 2013). However, in western countries, researchers are still doubtful the effectiveness of SFI. Thus, the present meta-analysis was conducted in an attempt to assess the effect of SFI on breast cancer patients.

A total of 49 studies were included in the meta-analysis, which we believed to be the largest one by far on this topic. Results showed that, with the help of the SFI, the objective tumour response rate and performance status were significantly improved, indicating a better curative effect and quality of life in patients treated with chemotherapy plus SFI. The antitumour mechanism of SFI is still unclear. Research suggested that SFI could inhibit the proliferation of tumour cells by inducing G2/M cell cycle arrest (Tin et al. 2007). The introduction of chemotherapy significantly improved the survival of breast cancer patients. However, the acquired drug resistance might weaken the treatment effect. Previous study also reported that SFI could re-sensitized chemotherapy to tumour cells through the initiation of mitochondrial apoptosis (Xiong et al. 2018). Based on the results of this meta-analysis, SFI could be recommended as complementary treatment for breast cancer patients.

The immune system is the frontline of defence against cancer in human and eliminates cancer cells from normal tissues. However, chemotherapy has cytotoxic to normal cells and can further induce immunodepression as chemotherapy unselectively target both cancer and normal cells. SFI was found to be an effective solution for repairing the immunity of cancer patients, alleviating chemotherapy induced immunosuppression and prolonging patients survival (Yang et al. 2017). It was suggested that the increases of NK, CD3^+^, CD4^+^ and CD4^+^/CD8^+^ ratio and the decrease of CD8^+^ levels represented the improvement of immunosuppressive status (Yang et al. 2017). Results from our study demonstrated that SFI intervention had positive effect on enhancing immune function and alleviating the damage caused by chemotherapy with enhanced NK, CD3^+^, CD4^+^ levels and CD4^+^/CD8^+^ ratio. Sensitivity analysis was performed with NK, CD3^+^, CD4^+^, CD8^+^ data due to high *I*^2^ values, and the study of Nie was suspected to be the source of heterogeneity. By comparing the study design of Nie with other included studies, we found that Nie et al. (2005) utilized a NVB chemotherapy regimen and SFI was only given 7 d for only one cycle, which is much shorter than others. The treatment duration of SFI and the combination of SFI with different chemotherapy might influence its effect on breast cancer patients. Further research is needed concerning these aspects.

The concomitant toxicity and adverse side effects is another unsolved problem related to the use of chemotherapy. Some complications not only have a negative impact on physical and metal healthy, but also are predictors of cancer patients’ survival. Therefore, it is urgently needed to reduce the incidence of chemotherapy-related side effects. The detoxication of SFI combined with chemotherapy was also proven in our study. Data showed that SFI intervention played an important part in reducing incidence of leucopenia, thrombocytopenia, haemoglobin reduction, liver dysfunction, gastrointestinal reaction, nausea and vomiting, bone marrow suppression and ECG changes. The effect of SFI on renal disorder required larger sample size analysis.

The findings of our meta-analysis were consistent with a previous study. Lv et al. (2015) concluded that SFI combined with chemotherapy in treating breast cancer can enhance the immunity of patients and further improve the clinical efficacy and safety (Lv et al. 2015). However, the study of Lv only included 18 trails and each subgroup contained less than ten pairs of data which limited the credibility of the results. Our study enrolled up to 49 studies for data pooling, which would be favourable in a systematic review and meta-analysis (Gopalakrishnan and Ganeshkumar 2013). Also, meta-regression analysis based on chemotherapy regimen, timing of experiment and treatment duration was performed, and the three factors were proved to be irrelevant to the heterogeneity among studies.

Nevertheless, several limitations still existed in the current analysis. The inclusion of low-quality publications might introduce bias in the analysis. Quality assessment showed that most of the included studies scored less than three according to the Jadad scale. Among these low-quality studies, the description of detailed procedure of randomization, methods of allocation concealment and the use of blinding interpretation were missing, which might bring about selection bias and overestimation of the SFI treatment effect. Also, the Begg’s and Egger’s test demonstrated that there was publication bias among the included studies. Although both English and Chinese databases were searched, none of the English publications were included, which might induce language bias. Moreover, our study only involved breast cancer patients from Chinese, thus the conclusion might only be applicable for Chinese ethnicity. Whether SFI is effective in other ethnicities still need further investigation as different body structures, life styles or other factors might affect the performance of SFI.

## Conclusions

Based on the evidence of the meta-analysis, the application of SFI could improve curative effect of chemotherapy with increased objective response rate, enhanced immune function and reduced toxic side effects. Although our study revealed promising results of SFI in treating breast cancer, a lot is still unknown regarding the mechanism, dosage, treatment duration and induced side effects. Therefore, the results of this meta-analysis should be interpreted with caution. However, we cannot ignore the fact that our meta-analysis still provides useful information of SFI in clinical practice. Further high quality, placebo-controlled and double-blinded clinical trials with larger sample size and proper randomization and allocation concealment are required to verify our results.

## Data Availability

All data generated or analysed during this study are included in this published article.
